# Understanding the diagnostic yield of current endoscopic biopsy for gastric neoplasm: A prospective single-center analysis based on tumor characteristics stratified by biopsy number and site: Erratum

**DOI:** 10.1097/01.md.0000505543.32353.f3

**Published:** 2016-10-21

In the article “Understanding the diagnostic yield of current endoscopic biopsy for gastric neoplasm: A prospective single-center analysis based on tumor characteristics stratified by biopsy number and site”,^[1]^ which appeared in Volume 95, Issue 30 of *Medicine*, the authors’ names were originally not given in full. The first affiliation was also not given completely. The article has since been corrected online.
